# microRNA-155-3p delivered by M2 macrophages-derived exosomes enhances the progression of medulloblastoma through regulation of WDR82

**DOI:** 10.1186/s12967-021-03156-y

**Published:** 2022-01-04

**Authors:** Li Song, Bin Luan, Qingrong Xu, Ruihe Shi, Xiufang Wang

**Affiliations:** Department of Pediatric Respiratory Medicine, The 3rd Affiliated Hospital of Zhengzhou University, No. 7, Kangfuqian street, Zhengzhou, 450052 Henan China

**Keywords:** Medulloblastoma, M2 macrophages, Exosomes, MicroRNA-155-3p, WD repeat domain 82, Proliferation, Invasion, Migration, Apoptosis

## Abstract

**Objective:**

Exosomes, membranous nanovesicles, naturally bringing proteins, mRNAs, and microRNAs (miRNAs), play crucial roles in tumor pathogenesis. This study was to investigate the role of miR-155-3p from M2 macrophages-derived exosomes (M2-Exo) in promoting medulloblastoma (MB) progression by mediating WD repeat domain 82 (WDR82).

**Methods:**

miR-155-3p expression was detected by RT-qPCR. The relationship of miR-155-3p with clinicopathological features of MB patients was analyzed. M2-Exo were isolated and identified by TEM, NTA and Western blot. CCK-8 assay, colony formation assay, flow cytometry, wound healing assay, and Transwell assay were performed to explore the role of miR-155-3p-enriched M2-Exo on the progression of MB cells. Luciferase assay were used to identify the relationship between miR-155-3p and WDR82. The effect of miR-155-3p-enriched M2-Exo on tumorigenesis of MB was confirmed by the xenograft nude mice model.

**Results:**

miR-155-3p was up-regulated in MB tissues of patients and MB cell lines. High miR-155-3p expression was correlated with the pathological type and molecular subtype classification of MB patients. WDR82 was a direct target of miR-155-3p. miR-155-3p was packaged into M2-Exo. miR-155-3p-enriched M2-Exo promoted the progression of Daoy cells. miR-155-3p-enriched M2-Exo promoted in vivo tumorigenesis.

**Conclusion:**

The study highlights that miR-155-3p-loaded M2-Exo enhances the growth of MB cells via down-regulating WDR82, which might provide a deep insight into MB mechanism.

**Supplementary Information:**

The online version contains supplementary material available at 10.1186/s12967-021-03156-y.

## Introduction

Medulloblastoma (MB) is a most prevalent malignancy in children [1]. The occurrence of MB is the highest in brain tumors, while its prognosis is much unpleasing [2, 3]. MB is assigned into four main molecular subgroups, including wingless, sonic hedgehog, Group 3 and Group 4, each with mutational and transcriptomic signatures, clinical outcomes and apparent cytogenetics [4]. Risk factors of MB consist of radiation, age at diagnosis, female sex and additional unknown elements [5]. High-dose chemotherapy, surgery resection and radiation to the primary tumor site and craniospinal axis are the universal therapeutic approaches for MB [6]. However, the 5-year overall survival rate of patients with MB is around 65% [7], and the survivors have to endure neurological, cognitive and endocrine disorders resulted from the aggressive therapy [8]. Therefore, it is essential to develop more feasible and effective therapeutic strategies for MB.

MicroRNAs (miRNAs) control biological processes [9]. A previous study has demonstrated that miR-155 takes on a crucial role in accelerating glioma progression and offers prognostic values [10]. In addition, miR-155-3p suppression could reduce glioma cell growth and proliferation in a mouse model and elevates the survival of mice with gliomas [11]. Macrophages are inborn immune cells that could modify different kinds of immune responses, antigen presentation and phagocytosis. After differentiation from their monocyte precursor, macrophages polarize toward M1-like or M2-like phenotype [12]. Exosomes, an important form of extracellular vesicles, have been confirmed as the intercellular communication mediators in various physical processes related to cell proliferation and migration [13]. Exosomes can be defined by several common characteristics, including size, density, morphology, and certain enriched protein markers [14]. Exosomes can transfer macrophage phenotype to M2 activated macrophages [15]. It has been suggested that exosomes are involved in MB tumor biology, such as stimulation of tumor cell proliferation and migration [16]. In the present study, we first characterized the exosomes to determine their size, appearance and expression of protein exosomal markers, in accordance with recommendations by minimal information for studies of extracellular vesicles 2018 (MISEV2018) [17], and then explored that whether M2 macrophages-derived exosomes (M2-Exo) could load miR-155-3p to mediate the progression of MB cells. M2-Exo display high miR-155 expression, and M2-Exo-mediated tumor growth is partly depended on miR-155 [18, 19]. WD repeat domain 82 (WDR82) is a c-terminal domain-combination protein recruiting the Setd1A Histone H3-Lys4 methyltransferase complex [20]. It has been accepted that WDR82 reduction is correlated with a poor prognosis of patients with colorectal cancer (CRC) [21], while the relationship of WDR82 with MB was little studied.

Therefore, the aim of this study was to investigate the role of miR-155-3p-loaded M2-Exo in MB progression by regulating WDR82.

## Materials and methods

The experiments were approved by the Institutional Review Board of the 3rd affiliated hospital of Zhengzhou University. All participants provided written informed consent. Efforts were made to avoid all unnecessary distress to the animals.

### General information of patients

The tissue samples of 79 MB patients were collected from the 3rd affiliated hospital of Zhengzhou University. The median age at diagnosis for MB patients was 9.94 years. Inclusion criteria: complete clinical data; MB confirmed via the pathologic diagnosis; no radiotherapy or chemotherapy before surgery. Exclusion criteria: incomplete clinical data; other primary tumors. Another 20 cases of normal cerebellar tissues were taken as a control via surgery excision after cerebellar hemorrhage.

### Immunohistochemistry

Paraffin-embedded sections were deparaffinized in xylene and rehydrated in 100, 95, 90, 85, and 75% gradient series of ethanol. Then, the antigen was repaired in a citrate buffer (pH 6.0) at 120 °C, the endogenous peroxidase activity was blocked with 3% H_2_O_2_, and the sections were incubated with WDR82 primary antibody (1:200, Abcam, MA, USA) overnight and with the secondary antibody (1:500; Abcam). Diaminobenzidine-developed sections were counter-stained with hematoxylin solution.

### Extraction and induction of bone marrow-derived macrophages

BALB/c mice (Henan Experimental Animal Center, Henan, China) aging 6–8 weeks, weighing 18–20 g, were euthanized. The femur and tibia were taken, and the bone marrow was syringed by serum-free Roswell Park Memorial Institute (RPMI) 1640 medium and filtrated with a 70-μm mesh. The filtrate was centrifuged and lysed with 5 mL erythrocyte lysis for 5 min. The monocytes obtained were stimulated in RPMI 1640 medium containing macrophage colony-stimulating factor (M-CSF, Peprotech, 25 ng/mL) for 72 h, 48 h, and 24 h (the medium was renewed at each time point), thus to differentiate to M0 macrophages.

To generate M2 macrophages, M0 macrophages were incubated with 20 ng/mL interleukin (IL)-4 (Peprotech) and 20 ng/mL IL-13 (Peprotech) for 24 h. Cell morphology was observed under an inverted microscope, and M2 macrophages-related markers arginase1 (AGR1) and CD206 were analyzed by Western blot.

### M2 macrophage transfection and exosome purification and characterization

Isolated M2 macrophages were transfected with miR-155-3p mimic or mimic NC (Ribobio, Guangzhou, China) via lipofectamine 3000 (Invitrogen, CA, USA). The ultracentrifugation method was used to extract exosomes from M2 macrophages transfected with miR-155-3p mimic or mimic NC and named Exo, Exo-miR-155-3p mimic and Exo-mimic NC accordingly.

Exosomes were isolated from the culture supernatant of M2 macrophages with ultracentrifugation method. The collected culture supernatant was centrifuged at 500×*g* and at 2000×*g* to remove the cell precipitation and cell debris. The obtained solution was filtrated via a 0.22-μm membrane and centrifuged at 1,00,000×*g*. The precipitation was re-suspended with PBS and centrifuged at 1,00,000×*g* to obtain exosome precipitation. During this process, the temperature of the sample never fell below 4 °C. The quantity of exosomes was measured using the BCA Protein Assay Kit (Beyotime, Shanghai, China). Western blot, nanoparticle tracking analysis (NTA) and transmission electron microscopy (TEM) were used to identify M2-Exo.

### Fluorescence microscopy analysis of exosome internalization

CM-Dil (2 μL, Sigma-Aldrich, MO, USA) was mixed with 100 μg exosomes, and resuspended in 18 mL PBS for 2-h centrifugation (1,20,000×*g*). The pellet was resuspended in 20 mL PBS and centrifuged at 1,20,000×*g* for 2 h. Then, the pellet was resuspended in 200 μL PBS and incubated with cells for 24 h. After fixation with polyformaldehyde, cells were observed under a fluorescence microscope.

### Cell culture and treatment

In MB cell lines Daoy, D283, ONS-76, D341 and human glial cells (Shanghai YaJi Biological, Shanghai, China), STR identification and mycoplasma detection were performed.

Daoy cells were cultured in Dulbecco’s Modified Eagle Medium (DMEM) containing 10% fetal bovine serum (FBS) and 1% penicillin–streptomycin mixture. Cells with 60% confluence were transfected using Lipofectamine 3000 (Invitrogen), and collected at 48 h after transfection for further experiments. Daoy cell line was transfected with miR-155-3p mimic (10 nM), mimic NC (10 nM), miR-155-3p inhibitor (25 nM), inhibitor NC (25 nM), miR-155-3p mimic (10 nM) + WDR82 overexpression plasmid (1 μg/mL), and miR-155-3p mimic (10 nM)  +  empty plasmid (1 μg/mL), respectively. All the oligonucleotides or plasmids were obtained from Ribobio (Guangzhou, China).

Five μg of Exo, Exo-miR-155-3p mimic and Exo-mimic NC were co-cultured with 1 × 10^5^ Daoy cells for 48 h when the cell confluence was 60%. Cells were collected for further experiments.

### Proliferation assay

Transfected Daoy cells or Daoy cells treated with M2-Exo were cultured in 96-well plates, and analyzed by CCK-8 (Dojindo, Japan). The optical density_450 nm_ value was detected on the micro-plate reader at 24 h, 48 h and 72 h.

### Colony formation assay

Transfected Daoy cells or Daoy cells treated with M2-Exo were seeded in 6-well plates with 200 cells/well [22]. The experiment was terminated 2 weeks later when the colonies were visible. The cells were fixed with anhydrous methanol solution, stained with 0.1% crystal violet solution, and photographed. The number of colonies was counted using the Image J software.

### Cell invasion assay

A 24-well Transwell plate (Corning) pre-coated with Matrigel (BD) was used for cell invasion. Transfected Daoy cells or Daoy cells treated with M2-Exo (4 × 10^4^) were seeded into the upper chamber. The medium in the upper chamber was FBS-free DMEM, and the medium in the lower chamber was DMEM supplemented with 10% exosomes-free FBS. After 24 h, cells transferred to the lower chamber were stained with 0.5% crystal violet and photographed under a microscope (Nikon, Japan).

### Scratch test

Transfected Daoy cells or Daoy cells treated with M2-Exo were seeded with 5 × 10^4^ cells per well into 24-well plates. After cell adherence, a straight line was drawn on the monolayer cells using a 10 μL pipette. Cells were cultured with FBS-free DMEM (500 μL/well) for 24 h and observed under an inverted microscope.

### Flow cytometry

Transfected Daoy cells or Daoy cells treated with M2-Exo were seeded into 6 well plates with 1 × 10^6^ cells/well. After culturing for 12 h, cells were resuspended with 100 μL buffer. According to the protocol of Annexin V-Fluorescein Isothiocyanate (FITC) Apoptosis Detection Kit (Beyotime), 10 μL FITC (50 mg/L) and 5 μL propidium iodide(50 mg/L) were added to the cultured cells. Then, cells were added with 200 μL binding buffer and loaded to the flow cytometer (FACSCalibur, BD Biosciences, NJ, USA).

### In vivo experiment

BALB/c nude mice of specific pathogen-free grade, aging 3–4 weeks, were purchased from Henan Experimental Animal Center. To observe the effect of miR-155-3p on tumor growth in MB, Daoy cells (1 × 10^6^) transfected with miR-155-3p overexpression lentivirus, miR-155-3p low expression lentivirus were suspended in 100 µL PBS and injected subcutaneously in the left groin of nude mice. To observe the effect of M2-Exo on the growth of MB, Daoy cells (1 × 10^6^) were subcutaneously injected into the left groin of nude mice. Meanwhile, 5 mg exosomes (Exo, Exo-miR-155-3p mimic and Exo-mimic NC) were administered into mice via tail vein injection once every 3 days for 2 weeks. The tumor volume (V  = 1/2 × L  ×  W^2^, L  =  tumor length, W  =  tumor width) was measured every 7 days with a vernier-caliper. A tumor growth curve was drawn and all nude mice were euthanized after 28 days.

### RT-qPCR

Total RNAs were isolated form tissues, cells, exosomes using Trizol (Invitrogen), and A260/A280 was determined by an ultraviolet spectrophotometer. RNA concentration (μg/μL)  =  (A260 × 40 ×  dilution factor)/1000. The purity should be 1.8–2.1. For WDR82, cDNA was collected from RNA (2 μg) through first-strand cDNA synthesis kit (Thermo Fisher Scientific) while for miR-155-3p, that was collected through NCode miRNA first-strand cDNA kit (Invitrogen). Real-time PCR was performed on the ABI7900 PCR system (Applied Biosystems, CA, USA) using SYBR Green PCR Master Mix (Takara, Dalian, China). The loading control of miR-155-3p was U6, and that of WDR82 was β-actin. The relative expression was calculated by 2^−△△Ct^ method and the primer sequences are shown in Additional file 1: Table S1.

### Western blot analysis

The total protein was extracted from tissues, cells and exosomes using modified RIPA buffer and sonication, and the protein concentration was detected by BCA method. Total protein extract (20 µg) or exosomal protein (10 µg) was separated using a 10% or 15% polyacrylamide gel and transferred to a 0.22-μm polyvinylidene fluoride membrane (Merck Millipore, USA). The membrane was blocked with 5% skim milk for 1 h, and incubated with the primary antibodies WDR82 (1: 100), β-actin (1: 1000, Abcam), CD206 (1: 1000, R&D Systems, Minneapolis, MN, USA), ARG1 (1: 1000, Proteintech, Chicago, USA), CD81 (1:1000), Alix (1: 1000), TSG101 (1: 1000), GRP94(1:500) (Santa Cruz Biotechnology, CA, USA) overnight at 4 ℃, and the corresponding secondary antibody for 1 h. The membrane was reacted with enhanced chemiluminescence solution for 5 min and detected in the exposure apparatus.

### Dual luciferase reporter gene assay

The luciferase reporter assay was carried out using pmiR-RB-REPORT vector (RiboBio) containing the wild-type (WT) or mutant (Mut) WDR82 3′-UTR sequences. miR-155-3p mimic or the corresponding controls along with the WT/Mut WDR82 3′-UTR vectors was transfected into Daoy cells using Lipofectamine 3000 (Invitrogen). At 48 h after transfection, the dual luciferase assay kit (Beyotime) was used to measure luciferase activity; the luciferase activity was standardized to Renilla luciferase activity.

### Statistical analysis

Statistical analyses were performed with SPSS 21.0 (IBM, Chicago, IL, USA) and Graphpad Prism 6.0 (GraphPad Software, La Jolla, CA, USA). Data were presented as mean  ±  standard deviation (SD). Student’s t test was applied to evaluate the significance between two samples. analysis of variance (ANOVA) was used for comparison among multiple groups, and Tukey’s post hoc test was applied for pairwise comparison after ANOVA. Correlation analysis was conducted by Pearson test. The correlation between miR-155-3p expression and the clinicopathological characteristics of patients with MB was determined via chi-square test or Fisher’s exact test. Predictors were kept if they were significant at a *P* value of 0.05 or smaller.

## Results

### Increase in miR-155-3p level in MB tissues; correlation of miR-155-3p levels with the clinicopathological characteristics of MB

**Fig. 1:**
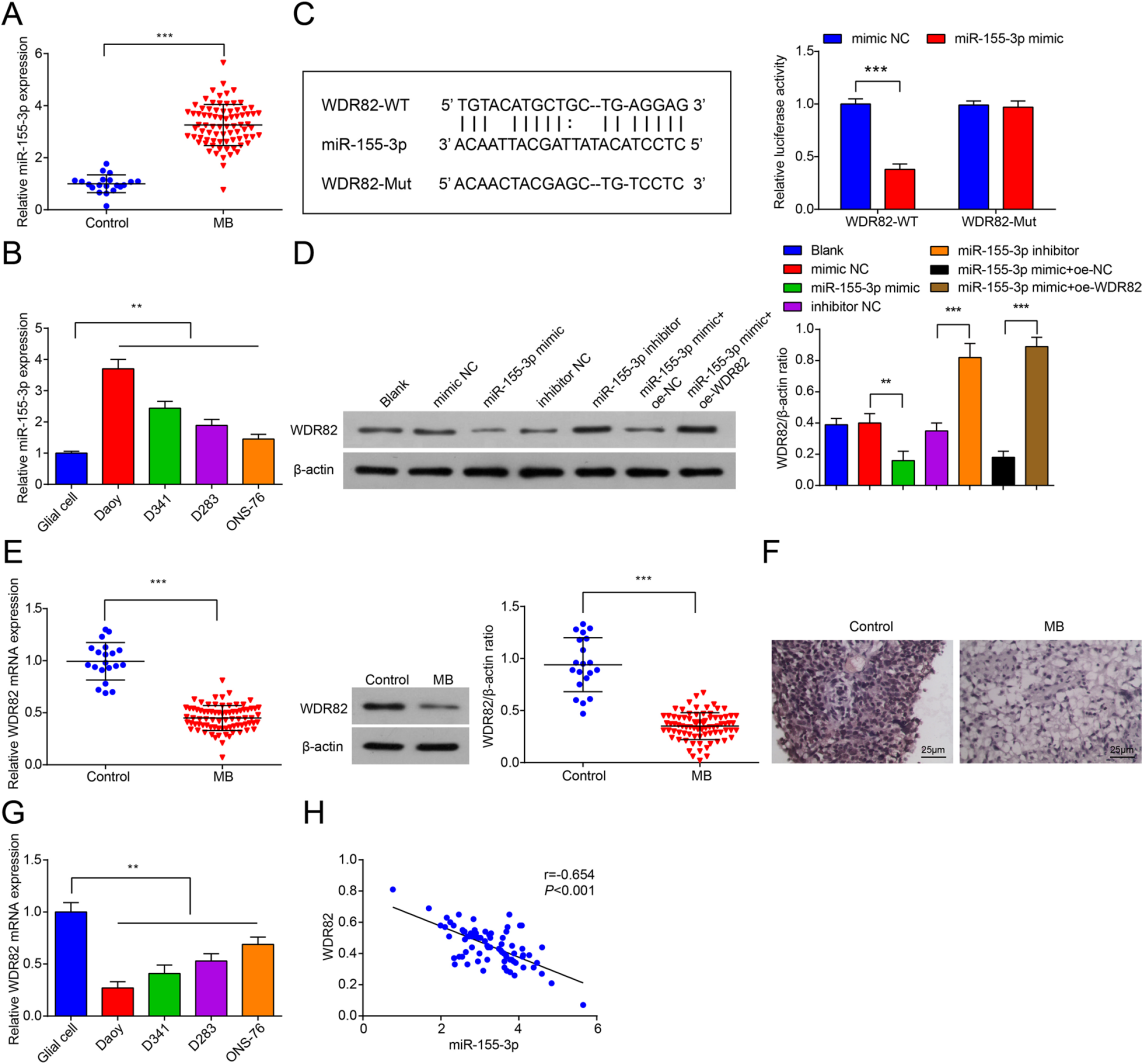
Increase in miR-155-3p level in MB tissues. **A** MiR-155-3p expression in MB tissues (n  = 79) and normal cerebellar tissues (n  = 20); **B** MiR-155-3p expression in Daoy, D341, D283, ONS-76 cells and glial cells; **C** a dual-luciferase reporter showed significant reduction of luciferase activity of the wild-type, and luciferase activity was restored by the mutant sequence; **D** WDR82 levels in Daoy cells after transfection; **E** WDR82 expression in MB tissues (n  = 79) and normal cerebellar tissues (n  = 20); **F** WDR82 immunohistochemical staining in MB tissues and normal cerebellar tissues (× 400); **G** WDR82 expression in Daoy, D341, D283, ONS-76 cells and human glial cells; **H** the correlation of miR-155-3p and WDR82 mRNA expression in MB tissues. Data were presented as mean  ±  SD. Statistical analysis was by Student’s t test or one-way ANOVA, Pearson analysis was conducted to determine the correlation between miR-155-3p and WDR82 mRNA expression. **P  < 0.01; ***P  < 0.001

To analyze the function of miR-155-3p in MB, miR-155-3p expression in MB tissues was detected by RT-qPCR and the Ct values of each samples were converted to fold changes using 2^−ΔΔCt^ method and then compared with the fold changes of the control group. The mean Ct value for miR-155-3p in the control group was 26.34. Based on our results, miR-155-3p expression was elevated in MB tissues (Fig. 1A). The relationship between miR-155-3p and clinicopathological characteristics of patients with MB was analyzed. Taking the median expression of miR-155-3p as the cutoff, the MB patients were assigned into low expression and high expression groups. chi-square test or Fisher’s exact test manifested that the pathological type and molecular subtype classification of MB were correlated with miR-155-3p expression, while age, gender, tumor location, metastatic status and Residual tumor size were not (Table 1).

**Table 1:** The relationship between miR-155-3p expression and the clinicopathological characteristics of patients with MB

Clinicopathological characteristics	N	miR-155-3p expression		*P*
		Low expression	High expression	
		(n = 39)	(n = 40)	
Age (years)				0.422
< 10	62	29	33	
≥ 10	17	10	7	
Gender				0.494
Male	48	22	26	
Female	31	17	14	
Tumor site				0.344
Cerebellar vermis	53	24	29	
Cerebellar hemisphere	26	15	11	
Pathological type				0.031
Classic type	46	28	18	
Anaplastic/large cell type	20	8	12	
Fibrogenic/nodular type	13	3	10	
Molecular subtype				0.005
Wnt	26	18	8	
SHH	14	9	5	
Non-Wnt/SHH	39	12	27	
Metastatic status				0.261
M0	43	24	19	
M1–3	36	15	21	
Residual tumor size (cm^2^)				0.252
≤ 1.5	64	34	30	
> 1.5	15	5	10	

miR-155-3p expression in MB cell lines Daoy, D341, D283 and ONS-76 cells and human glial cells was examined by RT-qPCR and the Ct values of each samples were converted to fold changes using 2^−ΔΔCt^ method and then compared with the fold changes of human glial cells. Mean Ct value for miR-155-3p in human glial cells was 24.60. It was determined that miR-155-3p expression was higher in Daoy, D341, D283 and ONS-76 cell lines than human glial cells (Fig. 1B). Due to the high expression of miR-155-3p in Daoy cells, this MB cell line was used for subsequent experiments.

### miR-155-3p directly targets WDR82 in MB cells

The bioinformatics analytical tool RNA22 was used to understand potential target genes of miR-155-3p in MB. WDR82 3′-UTR and miR-155-3p sequences were matched, as confirmed by transfecting the luciferase reporter containing WDR82 3′-UTR in Daoy cells. It was observed that miR-155-3p mimic reduced the relative luciferase activity of WT-WDR82 by nearly 62%, while the inhibition observed in cells transfected with Mut-WDR82 luciferase was minimal (Fig. 1C).

WDR82 expression in Daoy cells that overexpressed or underexpressed miR-155-3p was further tested. It was noticed that miR-155-3p mimic reduced WDR82 expression while miR-155-3p inhibitor up-regulated WDR82 expression in Daoy cells. Daoy cells were transfected with miR-155-3p mimic, in the absence or presence of WDR82 plasmid for more clarity on the synergistic effects of WDR82. It was found that in Daoy cells, overexpression of WDR82 reversed the decrease in WDR82 expression caused by miR-155-3p mimic (Fig. 1D).

Our findings also showed WDR82 expression was suppressed in MB tissues (Fig. 1E, F). RT-qPCR and Western blot analysis also determined that WDR82 expression was lower in Daoy, D341, D283 and ONS-76 cell lines than human glial cells (Fig. 1G). Next, Pearson’s correlation analysis in MB tissues revealed an inverse relationship between WDR82 and miR-155-3p expression (Fig. 1H). Table 2 details the expression of miR-155-3p and WDR82 in MB tissue and normal cerebellum tissue.

**Table 2:** Expression of miR-155-3p and WDR82 in MB tissues and normal cerebellar tissues

Markers	Normal cerebellar tissues	MB tissues
miR-155-3p	Low	High
WDR82	High	Low

### Inhibition of miR-155-3p impairs MB cell growth in vitro

**Fig. 2:**
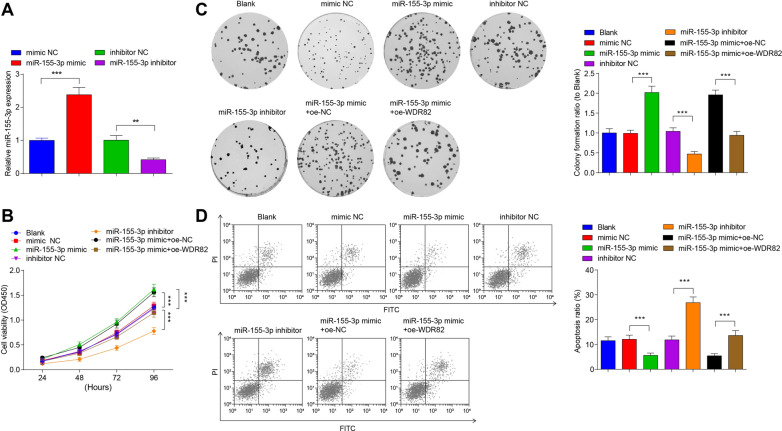
Inhibition of miR-155-3p impairs MB cell growth in vitro. **A** miR-155-3p levels in Daoy cells after transfection; **B** the proliferation ability of Daoy cells in each group; **C** the colony formation ability of Daoy cells in each group; **D** apoptosis of Daoy cells in each group; data were expressed in mean  ±  SD. Statistical analysis was by ANOVA; **P  < 0.01; ***P < 0.001

To evaluate the effect of miR-155-3p on MB cells, Daoy cells were transfected with miR-155-3p mimic or inhibitor to regulate miR-155-3p expression. miR-155-3p expression in each transfected Daoy cells was detected by RT-qPCR and the Ct values of each samples were converted to fold changes using 2^−ΔΔCt^ method and then compared with the fold changes of the mimic NC group. The mimic NC group mean Ct value for miR-155-3p was 28.16. Cell transfection with miR-155-3p mimic elevated miR-155-3p expression while transfection with miR-155-3p inhibitor declined miR-155-3p expression (Fig. 2A). The proliferation and colony formation abilities of Daoy cells were detected via CCK-8 and colony formation assays (Fig. 2B, C). Proliferation and colony formation abilities of Daoy cells were enhanced by transfection with miR-155-3p mimic while depressed by that with miR-155-3p inhibitor. The activities of miR-155-3p on cell proliferation and cell colony formation were depressed by overexpressing WDR82.

Cell apoptosis detected by flow cytometry indicated that miR-155-3p mimic transfection reduced while miR-155-3p inhibitor transfection heightened apoptosis rate of Daoy cells. WDR82 overexpression impaired miR-155-3p overexpression-induced apoptosis reduction (Fig. 2D).

### Reduced miR-155-3p represses invasion and migration abilities of MB cells

**Fig. 3:**
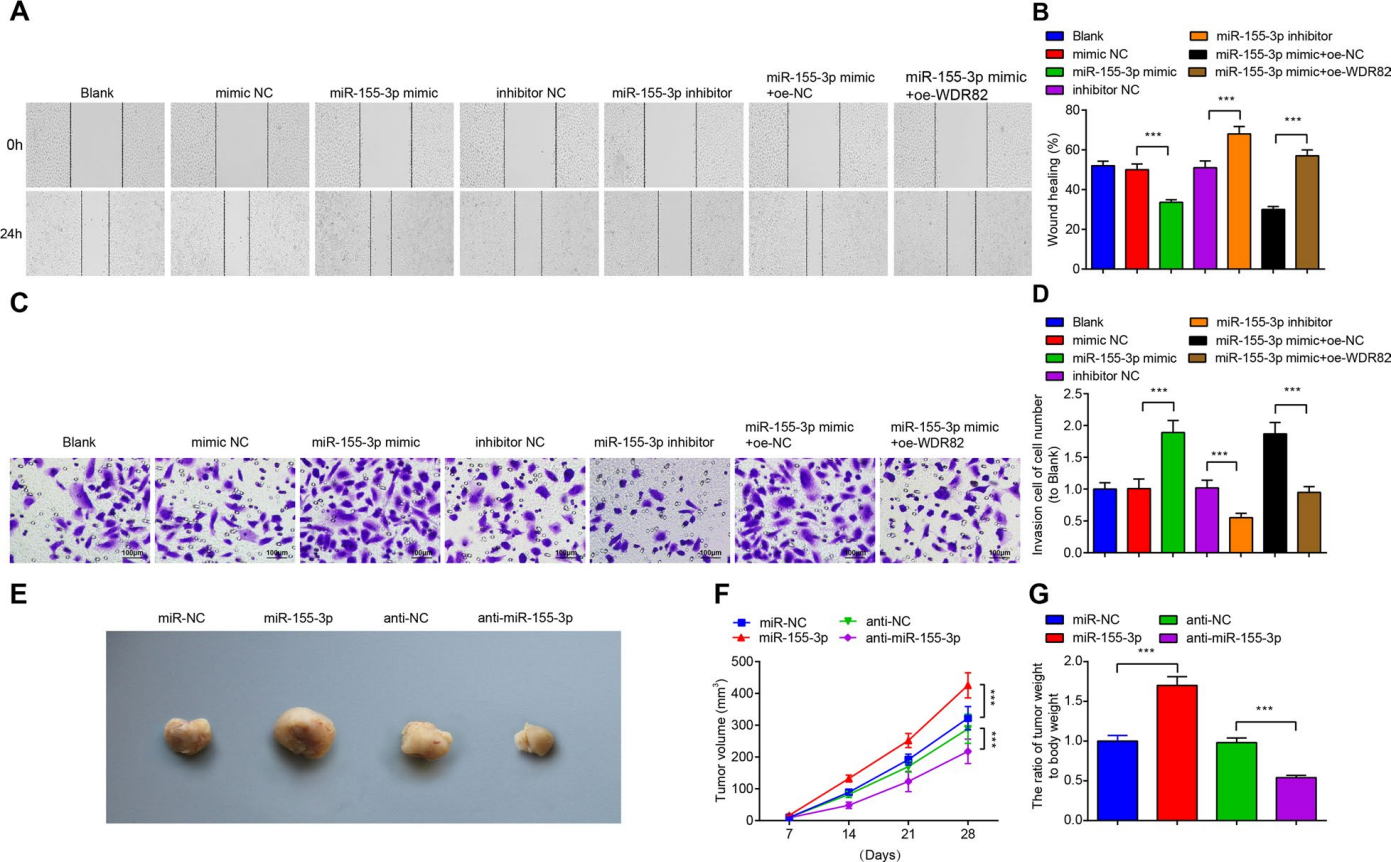
Inhibition of miR-155-3p impairs MB cell invasion and migration abilities, and inhibits MB cell growth in vivo. **A**, **B** The migration ability of Daoy cells in each group; **C**, **D** the invasion ability of Daoy cells in each group; **E** representative figure of tumors formed; **F** tumor growth curves in nude mice of each group; **G** tumor weight in nude mice of each group; data were expressed in mean  ±  SD. Statistical analysis was by ANOVA; ***P  < 0.001

Measurements of cell invasion and migration abilities demonstrated that miR-155-3p mimic transfection promoted invasion and migration of Daoy cells, while miR-155-3p inhibitor transfection worked in an opposite way; the activities of miR-155-3p on cell invasion and migration were inhibited by overexpressing WDR82 (Fig. 3A–D).

### Down-regulation of miR-155-3p inhibits MB cell growth in vivo

To further explore whether miR-155-3p influences tumor growth in vivo, we constructed Daoy cells with miR-155-3p overexpression or low expression and subcutaneously injected them into nude mice. After 28 days, the tumor volumes and weight were high in miR-155-3p-overexpressing Daoy cells. The tumor volumes and weight were lower in miR-155-3p low expression-modified Daoy cells (Fig. 3E–G).

### Identification of M2 macrophages and M2-Exo

**Fig. 4:**
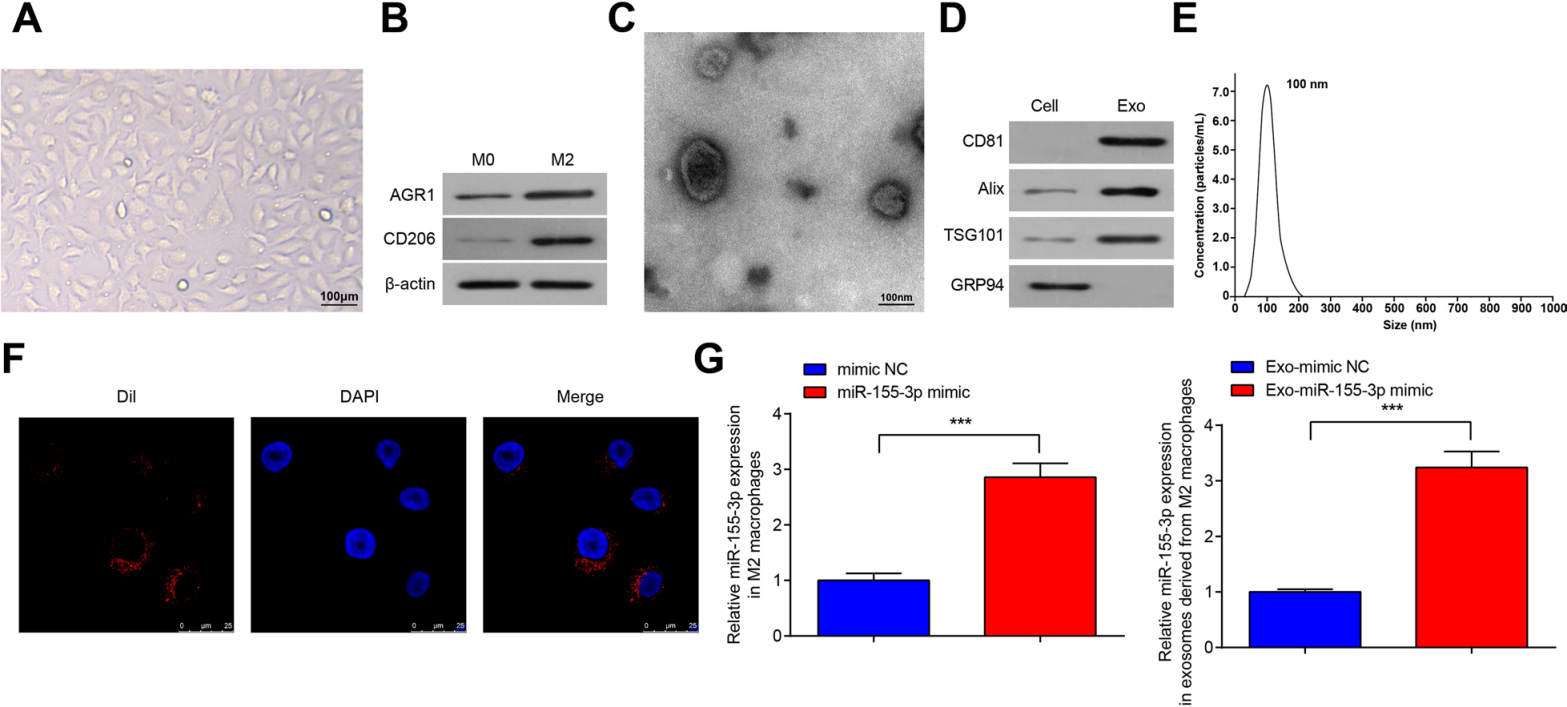
Identification of M2 macrophages and M2-Exo. **A** The morphology of M2 macrophages under an inverted microscope; **B** AGR1 and CD206 protein bands in M2 macrophages; **C** the morphology of M2-Exo under a transmission electron microscope; **D** CD81, Alix, TSG101 and GRP94 protein bands; **E** NTA of M2-Exo; **F** M2-Exo could be uptaken by Daoy cells; **G** miR-155-3p expression in M2 macrophages or M2-Exo. Data were reported as mean  ±  SD. Statistical analysis was by One-way ANOVA; ***P  < 0.001

M2 macrophages were identified. Under the microscope, M2 macrophages were mainly elongated (Fig. 4A). The markers of M2 macrophages were detected by Western blot, and it was detected that ARG1 and CD206 were up-regulated in M2 macrophages (Fig. 4B).

The exosomes isolated from M2 macrophages were identified by TEM, NTA and Western-blot. The results showed that the exosomes isolated from M2 macrophages had the following characteristics: morphology (bilayered membrane “round-shaped” vesicles), size (diameter 30–100 nm), and presence of exosomal positive protein marker (CD81, ALIX, TSG101) and non exosomal marker like the endoplasmic reticulum protein GRP94 (Fig. 4C–E).

To demonstrate that M2-Exos could be taken up by Daoy cells, we labeled exosomes with Dil, a membranal fluorescent carbocyanine dye, and found that Dil-labeled M2-Exos were taken up by Daoy cells after 24 h co-culture (Fig. 4F).

We detected miR-155-3p expression in M2 macrophages transfected with miR-155-3p mimic and mimic NC and in exosomes derived from M2 macrophages transfected with miR-155-3p mimic or mimic NC by RT-qPCR, and the Ct values of each samples were converted to fold changes using 2^−ΔΔCt^ method and then compared with the fold changes of the mimic NC group or Exo-mimic NC group. The mean Ct value for miR-155-3p of mimic NC group was 29.35 while that for miR-155-3p of Exo- mimic NC group was 28.22. The results showed that, in M2 macrophages, miR-155-3p mimic increased miR-155-3p expression. miR-155-3p expression was higher in exosomes derived from M2 macrophages transfected with miR-155-3p mimic than exosomes derived from M2 macrophages transfected with mimic NC (Fig. 4G).

### miR-155-3p-enriched M2-Exo accelerates cancer progression of MB cells

**Fig. 5:**
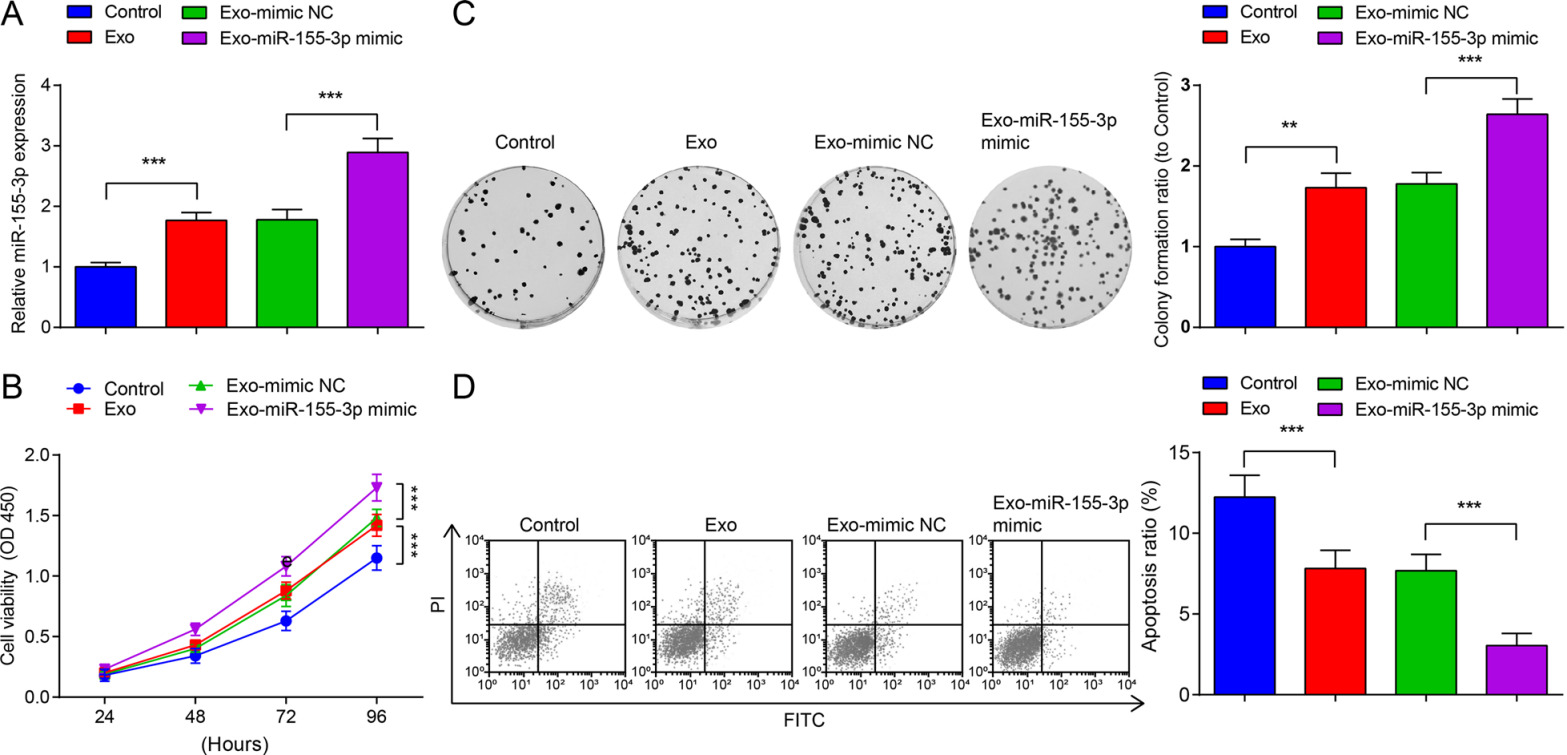
miR-155-3p-enriched M2-Exo accelerates MB cell growth in vitro. **A** miR-155-3p levels in Daoy cells after M2-Exo treatment; **B** the proliferation ability of Daoy cells in each group; **C** the colony formation ability of Daoy cells in each group; **D** apoptosis of Daoy cells in each group; data were shown as mean  ±  SD. Statistical analysis was by ANOVA; **P  < 0.01; ***P  < 0.001

**Fig. 6:**
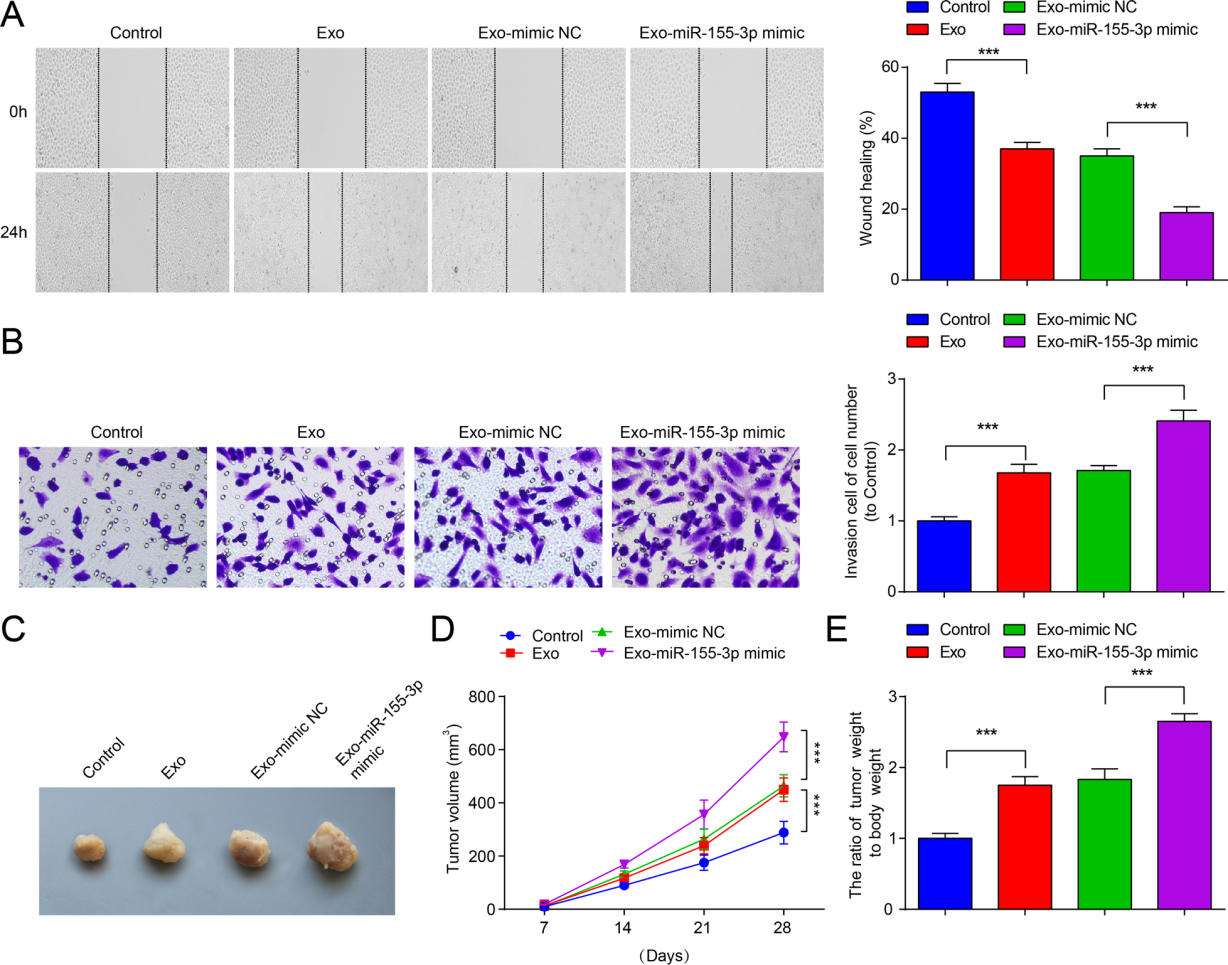
miR-155-3p-enriched M2-Exo enhance invasion and migration abilities of MB cells, and accelerates tumorigenesis of MB cells in vivo. **A** The migration ability of Daoy cells in each group; **B** the invasion ability of Daoy cells in each group; **C** representative figure of tumors formed; **D** tumor growth curves in nude mice of each group; **E** tumor weight in nude mice of each group; data were expressed as mean  ±  SD. Statistical analysis was by ANOVA; ***P  < 0.001

We observed the effects of M2-Exo and miR-155-3p-modified M2-Exo on the growth of MB cells in vitro. Firstly, miR-155-3p was checked by RT-qPCR in Daoy cells treated with M2-Exo, and the Ct values of each samples were converted to fold changes using 2^−ΔΔCt^ method and then compared with the fold changes of the Control group. The mean Ct value for miR-155-3p of Control group was 27.74. The test result showed that M2-Exo treatment increased miR-155-3p expression in Daoy cells. Exo-miR-155-3p mimic treatment increased miR-155-3p levels in Daoy cells compared to Exo-mimic NC treatment (Fig. 5A). The proliferation, colony formation, invasion, migration and apoptosis of Daoy cells after M2-Exo treatment were detected (Figs. 5B–D, 6A, B). Exo promoted the proliferation, colony formation, invasion and migration activities while suppressed apoptosis of Daoy cells. Exo-miR-155-3p mimic treatment had the enhanced effects on Daoy cells.

### miR-155-3p-enriched M2-Exo accelerates tumorigenesis of MB cells in vivo

To further explore the effects of miR-155-3p-enriched M2-Exo in MB, xenograft nude mice model was set up. Daoy cells were subcutaneously injected into nude mice, and 5 mg exosomes was administered into nude mice via tail vein injection once every 3 days for 2 weeks. The results showed that administration of Exo accelerated tumour growth, and the administration of Exo-miR-155-3p mimic further promoted tumor growth and tumor weight (Fig. 6C–E).

## Discussion

MB is a high-ranking malignancy recognized as a member of childhood cerebellum tumors [23]. Meanwhile, the pathophysiological basis of MB still remains poorly understood. Exosomes, virus-sized membrane vesicles produced extracellularly from cells may exhibit roles in MB pathogenesis but are as yet largely studied in this disease. For these reasons, this study was to investigate the role of the transfer of miR-155-3p by M2-Exo in promoting MB progression by targeting WDR82.

The observation of the study was that M2-Exo promoted the proliferation, colony formation, invasion and migration abilities, inhibited apoptosis in vitro, and elevated the tumor growth rate in nude mice in MB. Indeed, it has been clarified that exosomes are involved in the pathogenesis of MB [16]. Significantly, exosomes from human gastric epithelial cells or serum exosomes from infected patients and mice clearly reduce endothelial functions with decrease of migration and proliferation [24]. This is consonant with the fact that human bone marrow mesenchymal stem cell-derived exosomes facilitate osteosarcoma cell invasion, proliferation and migration [25]. Furthermore, it has been observed that M2-Exo could induce colon cancer cell proliferation and invasion and reduce apoptosis [26]. Also, Yuan et al. have described that M2-Exo perform aggressively during the progression of oral squamous cell carcinoma cell growth through acting as a carrier of miR-31-5p [27].

Our study revealed that miR-155-3p was up-regulated in MB tissues of patients and was correlated with the molecular subtype classification of MB. It has been found that miR-155 suppression may be a functional anti-tumor choice for glioma [10]. In addition, miR-155 is overexpressed in nonfunctional pituitary adenomas samples and adenomas [28] and in HCC [29]. Meanwhile, we presented that inhibition of miR-155-3p reduced proliferation, colony formation, invasion and migration abilities and promoted apoptosis of MB cells in vitro. According to a report by Tao et al. it is suggested that silencing of miR-155-3p restrains the proliferation, invasion and migration of clear cell renal cell carcinoma cells [30]. Moreover, it has been specified that miR-155-3p up-regulation in breast cancer cells blocks cellular apoptosis [31]. On the other hand, miR-155-3p up-regulation in CRC could reduce the levels of WDR82, involving in the promotion of tumor progression [32]. Besides, miR-155-3p could induce the polarization of M2 macrophages and promote the progression of glioma [33].

Some new findings were observed in this study that WDR82 was down-regulated in MB tissues of patients and cell lines; and WDR82 overexpression reversed the promoting effects of miR-155-3p up-regulation on MB cell progression in vitro and in vitro. In the area of CRC, it is described that WDR82 is lowly expressed in cancer patients, and knockdown of WDR82 is related to shortend overall survival and poorer outcomes [21]. As to the role of WDR82 in the aggressiveness of tumors, Lei et al. have discussed that WDR82 downregulation enhances the growth of lung cancer cells [34].

In conclusion, the study stresses that inhibited miR-155-3p-loaded M2-Exo repress MB cell progression through down-regulating WDR82, which might offer a deep insight on MB-related mechanism and molecule-based mechanism may be a possible target for future therapies of MB. More researches should be under taken to verify the relationship of miR-155-3p with WDR82 in MB.

## Supplementary Information

**Additional file 1: Table S1.** The primer sequences of genes.

## Data Availability

The original contributions presented in the study are included in the article/Additional file, further inquiries can be directed to the corresponding author.
