# Supplementary material for: microRNA-155-3p delivered by M2 macrophages-derived exosomes enhances the progression of medulloblastoma through regulation of WDR82
Source: J Transl Med. 2022 Jan 4;20:13. doi: 10.1186/s12967-021-03156-y (PMC8728908; doi:10.1186/s12967-021-03156-y)
Supplement: Supplementary file 1 — Additional file 1: Table S1. The primer sequences of genes. [file 12967_2021_3156_MOESM1_ESM.docx]

**Supplementary Table 1** The primer sequences of genes

| Gene Name | Primer sequence (5’-3’) |
| --- | --- |
| miR-155-3p | F: 5’- CTCCTACATATTAGCATTAACA-3’ |
| U6 | F: 5’- CTCGCTTCGGCAGCACA-3’ |
| WDR82 | F: 5’-TGATGACTGACCCTGTTGCT-3’ |
|  | R: 5’-ATCCAATCCCACTATTATCTCAG-3’ |
| GAPDH | F: 5’- GCCATCACTGCCACCCAGAAGACTG -3’ |
|  | R: 5’- CATGAGGTCCACCACCCTGTTGCTG -3’ |

**Note:** F, forward; R, reverse; miR-155-3p, microRNA-155-3p; WDR82, WD repeat domain 82; GAPDH, glyceraldehyde-3-phosphate dehydrogenase.
